# Extraction of Teeth

**Published:** 1854-01

**Authors:** J. Taylor


*Extraction of Teeth.* By DR. J. TAYLOR. [Continued.]

In our last (see No. 4, Vol. VI, Register) we spoke of the extraction of the dens sapientiæ and molars of the inferior maxilla, and had given our mode of removing these teeth, when broken down to the edge of the alveoli. We would now only add that, although we generally cut away a portion of the process, as then described, yet there are cases where we prefer at once applying our forceps made with single beak—laying hold of the process, and with the sharp beak of the instrument cutting away that portion which covers the neck of the tooth down as low as the bifurcation of the roots. If the forceps' points are sharp and well adapted, they will effect this without any difficulty.

For the bicuspids of the left side, we have described a forceps, constructed similarly to that for the molar of the same side, only in its adaptation to the tooth, which is for the bicuspid forceps, single beaked and grooved out, to fit the neck of the tooth.

In the application of the forceps, our position is the same as before, to the right and back of my patient; and instead of the inward and outward motion for the loosening of these teeth, we make a rotary motion. It should be recollected, that the roots of these teeth are generally round, and pointed. The roots are also of tolerable length, and often the necks of the teeth small; so that a sudden inward or outward motion (and particularly an outward), will break the tooth. We are often told by dentists, that they are more apt to break these teeth than any others, and we believe the reason is, that they apply a wrong force. The same application of force should here be applied, as to the upper incisors, a slight turning of the tooth in the socket.

When these teeth break off to the margin of the alveoli, they often give much trouble in their removal. This is owing to the depth of root, and the unresisting alveolar processes which surround them. We have found when the break has taken place as low down as the edge of the alveolus, that a narrow beaked root forceps, which will merely embrace that portion of the process, which covers the labial and lingual portion of the root, where the process is most thin, and hence, give to the force of the instrument most readily that it is the best for their removal. We then cut the process with the forceps' blade, and remove the root at the same time. This shortens the operation, and as the outer process generally stands on a higher level than the inner, but little of the latter need be taken hold of; and hence, an inward motion should be made to effect the loosening of the root and the cutting away of the bone. It will be seen here, that a rotary motion is inadmissable. For the removal of the bicuspids of the right side, we have a hawk's-bill forceps. This, like the molar forceps for the same side, would be inadmissible for the inward and upward motion for the removal of these teeth; but fortunately here, this is not necessary, for the rotary motion is best adapted to loosen these teeth; and as the tooth is felt to give, an upward lift disengages it from its alveolus. If these should break, we use the root forceps as before—only take our position a little more in front of our patient; still holding the chin in our left hand, and standing to the right of our patient.

For the extraction of the cuspidati, we use generally, the same forceps, using that for the left side on the left, and that for the right on the right. The same motions are generally requisite; yet, the roots of these teeth are sometimes flattened, requiring more of the inward and outward motion.

For the extraction of the lower incisors, we use generally, our straight or curved-root forceps, standing on a stool behind our patient, and usually find that an outward and upward motion is all that is necessary. These roots being flattened, the rotary motion is not applicable. They are the easiest teeth of all the set to extract.

We ceme now to speak of the extraction of the teeth in the superior maxilla, and shall commence with the dens sapientiæ. This tooth is sometimes difficult to get at, yet there is not generally that difficulty experienced, as in the same teeth of the lower jaw.

They are sometimes, for the want of room, thrown almost out of the circle; generally without. The roots are smaller than of the molars of the same jaw, and sometimes curved backwards under the maxillary tuberosity. When this is the case, they are generally thrown obliqely against the posterior approximal face of the molar. When they stand erect and fair, and not decayed too much on their labial face, we prefer at once taking the forceps already described for the purpose, and having the head of our patient thrown back far on the head-rest of our operating-chair, we steady the head with our left arm and hand, raising sometimes the lip with our forefinger of the left hand, and apply our forceps high upon the tooth, we make an outward and downward motion. If the outward move should not loosen the tooth, an inward move should be made before the direct pull is made, to disengage the tooth from the socket. When these teeth incline inward, the inward motion should generally be made first. The direction of these teeth as they stand in the alveolar ridge, and as they press against the posterior molar, should always be observed, before the application of force for their removal. If the roots are curved backward, the force should be applied so as to force them backward, and outward, or backward, and inward, as the direction of the roots and pointing of the teeth indicates.

When these teeth are much decayed on their labial face, and the hold for the forceps' blade at this point injured or made uncertain, and there is soundness sufficient next the molar for the application of Physic's Elevator, we use this without any hesitation. Although this instrument is especially designed for these teeth of the lower jaw, yet, they apply here just as well. We had a case only a few weeks since, a gentleman, some sixty miles in the interior of the state, in attempting to have an upper dens sapientiæ removed by a dentist with the forceps, had his tooth broken. The break had taken place on a level with its alveolus, except a very small point next the molar. This point could not have been over a line or two at most, above the socket, yet, under the gum

When we first made an examination, we saw no chance for the removal of the tooth ; but in passing our gum-lancet back of the molar, we found a point which we determined to use if possible, in the extraction of the roots. The application of the elevator was made, pressing the points of the blades as far up as possible, and they caught the projecting point, and raised the roots entirely out of the socket without any difficulty whatever. Here was a case where the forceps had utterly failed, for they had been thoroughly tried by a skilful dentist, and we are satisfied, that without cutting away the process, or closing them in the forceps' blades, the tooth could not have been removed with them. For the want of an elevator, the patient had to come over sixty miles to have his tooth extracted.

For the extraction of the posterior molar, we use a pair of forceps already described, holding the head of the patient in the same manner, as for the extraction of the dens sapientiæ. The patient's face is turned towards us, and this is the case when we extract the teeth of either side. In applying the force for the removal of the tooth, we generally, first make an inward motion, and then an outward and downward movement. If the roots are not divergent, this will generally remove the tooth; but if the roots diverge very much, it may not be sufficient; and when this is the case, we generally find that the inward movement has been of no avail, and that the outward motion is the one which first starts the tooth. If then on examination of the tooth, we find from its large development and firmness of attachment, with an expanded alveolar ridge, that the roots are large, and apparently diverge, we make the first move an outward one, and the second inward and downward. If these teeth should hold after being loosened, a rocking and shaking of the tooth may be necessary for their removal from the alveolus.

For the removal of the anterior molar, we use generally, the same forceps as for the posterior molar; yet, if the tooth is more than ordinarily firm, and the roots divergent, we select a pair of larger and stronger forceps, made on purpose,

(but of the same pattern) and apply them. Some of these teeth require more force to extract than any others; and when we anticipate this, we place ourself a little farther off from our patient; still however, retaining our hold of the patient's head, but in this placing ourself farther off from the patient, we have more room for the play of the muscles of the arm, and can exert more force for the removal of the tooth.

We almost always make here the outward motion first, and this is made somewhat on a line with the direction of the palital root of the tooth. As we feel the tooth give, we draw inward and downward, and if the tooth holds in the socket after having been loosened, we rock and shake the tooth, still pulling downward, until it is removed from its alveolus.

In a few cases in the removal of the anterior molars of the left side, when more than ordinary force was requisite, we have taken our position on the left side of the patient, having the head well back on the head-rest, and the thumb of the left hand pressed against the palital face of the bicuspids of the same side, and the forefinger raising the lip, and against the labial face of these teeth; and as the tooth is inclosed in the forceps' blades, the head is steadied by the hold of the left hand, and the outward and downward movements are made with the forceps, for the removal of the teeth. This position gives an oportunity of exerting a little more force than any other, and some of these teeth require it. This position is one frequently taken by my brother, Dr. Joseph Taylor, and he tells me he was first driven to it in the removal of a tooth of this class for a gentleman of Kentucky, several years since, after having failed in attempting to take out the tooth by standing on the right side of the patient. When standing on the right side (as usual), he found, that to make the outward motion to loosen the tooth, the arm was thrown too much from the body, to exert the force necessary.

It may be in place here to make a few remarks, in relation to the use of the superior molar forceps, in taking out these teeth after they have been broken. The tooth may, and often does break just about the gum, and yet, not on a level with the alveolar processes; and when this is the case, it may only be necessary to re-apply the forceps, and secure a hold nearer the process; but should the tooth break close to the processes, then a different course is necessary, and we generally then pursue much the same practice, as recommended for the removal of the lower molars, when thus broken, that is, cut away a portion of the process on the labial part, and expose the roots where they unite, so as to permit the single pointed blade of the forceps, to take hold between the roots. The inner blade may inclose the edge of the process on the palital root, and act as the fulcrum, on which a leverage is applied to remove the roots. The outer blade passing between the labial roots at their bifurcation, hold very securely, and enable us to make a very powerful application of force inwardly and downward, for the extraction of the roots. In this operation we should expect the roots either to be removed or loosened and separated, so that they can be taken out separately with the root forceps. The practice of laying hold of the process and breaking it in with the forceps, although a quicker operation, yet, we only do it in refractory patients, when, on the breaking of the tooth, we can make the application, and secure its extraction, before the head is let up or the instrument taken out of the mouth.

For the removal of the bicuspids in the upper jaw, the instrument has been already described, and suits not only these teeth, but also, the cuspidati and incisors. It is true, for occasional use, we prefer a smaller-bladed forceps for the lateral incisor, but the strait root forceps generally answer for these, when too small for the others.

The most natural application of force for the extraction of the bicuspids, appears to be inward and downward. That is these teeth would appear to loosen easier by first an inward application of force. It will be recollected, that the roots of these teeth are flattened, and appear most generally, as two small roots united; sometimes separating at their points, and what is remarkable, the anterior more frequently separating than the posterior. The edge of the alveolar process is generally on a higher level on the labial than the palital side. There are cases, however, where the inward motion cannot well be made without catching the under lip between the teeth and bar of the forceps; and unless the teeth are frail, the outward and downward motion is generally sufficient. We make it a rule in the extraction of these teeth, when we feel the teeth sensibly move, by either an outward or inward motion, to pull downward, and somewhat reverse the outward or inward motion. Care, we think, should, however, be taken, not, to make these motions either too sudden, or too far from the direction of the alveolus; for if this is done, the danger of breaking the tooth is increased. In all frail teeth of this or any other class, it is far better to make repeated movements, only slightly moving the tooth, until it is felt to raise in the socket; and this should be done before the forceps are closed firmly enough for the direct removal of the tooth from the socket, and during this part of the operation, the forceps should be kept in close contact with the edge of the alveolar processes.

For the removal of the cuspidati and also the incisors of the upper jaw, a direct inward and outward motion is not necessary. It is true, the cuspidati roots, are occasionally, somewhat flattened; and when the rotary motion is not sufficient to loosen these teeth, an outward or inward motion is necessary; but this is not often.

The motion for the extraction of all these six front teeth, is what might be called a turning of the teeth in the socket; and as the tooth is felt to move, a downward pull is given for its removal.

In the use of the root forceps, there are only a few general directions necessary, bearing in mind the general form of the root to be removed, whether it be a round, conical shaped root or a flattened and sometimes curved one.

When the roots are frail and broken below the gum, we make careful examinations, to see if above the process, there is soundness and strength sufficient to bear the direct applicacation of the forceps' blade. We wish also to see if the alveolar processes adhere closely to the roots. If the roots are softened by decay, or frail in appearance, and the decay cupping down below the edge of the alveolus, we separate the gum well and force our gum lancet into the socket as far as practicable, and for this purpose our lancets are made slightly convex on one side and concave on the other, so as to fit on the neck of the teeth or of these roots, like a delicately formed forceps' blade. This forcing of the socket open is effected merely on the labial and palital portion, and is designed to permit the point of the forceps to pass in, and as the root gives, by the rocking, or the inward and outward motions, to secure a hold above the decay for its removal. Teeth that have been broken off for some time generally permit this procedure without much difficulty. The processes have either partially absorbed, or the alveole-dental membrane has thickened, and slightly raised the root in its alveolus.

In the application of the forceps, for the extraction of roots, we regard it, then, as of the first consequence that no sudden or very great application of force should be made, and that as each movement is made for the loosening of the root, the forceps should be forced, as it were, into the socket. The points of our root forceps' blades are kept sharp, and are often made, when the roots are not frail, to answer the purpose of gum lancets. They should enclose the root as a pair of moulds would that, which has been moulded in them. If properly tempered, they seldom if ever break by proper use. A few days since, we were requested to go several squares just before dusk, to extract a lower root for a lady, suffering very much with toothache, and unable to come to the office. Being assured it was simply an old root in the lower jaw, we took with us our root forceps and gum lancets. When we examined the mouth, we found, it is true, an old loose root of the dens sapientiæ tooth of the left side below, which was easily removed with a curved root forceps, and which was the most delicately formed one we had in our case. After its removal, the pain continued, and we made further examination, and found that the first large molar of the same side below, was the cause of the pain; being much decayed on its posterior approximal face. Being too late to return to our office, and get a molar forceps, we applied the root forceps; and although very firmly set, yet, it removed the tooth without any difficulty We allude to this case, merely to show the strength of what would be called a very small root forceps.

In the extraction of teeth with the forceps, or indeed with any other instrument, the more that can be well accomplished in a given and limited time, the better for the patient. Acting on this principle, and believing, that while an individual is suffering from the extraction of one tooth, the pain from the extraction of another is scarcely felt. We take out as many as possible, before the mouth is closed. We have thus, frequently, with the forceps, taken out from six to ten of the front, upper and under teeth, at one and the same operation, or before the patient closed their mouth, or stopped to spit. The bicuspids, cuspidati and incisors above, can all be removed with one forceps; so likewise, the same teeth below can be removed with the one forceps, and hence, all these teeth can be extracted without any change in the forceps, which would consume time in the operation.

We have laid down, perhaps more minutely than was necessary, the general directions for the construction and use of these instruments (the forceps), and in conclusion, would say, that when properly constructed and applied, they certainly are superior to all other instruments in use for the extraction of teeth. We shall, however, in another article, treat of such other instruments, as we occasionally use. These are most generally resorted to, when from peculiarity of situation, or something of the kind, the forceps cannot be well applied.

